# Comparative Study on Protein Composition and Foam Characteristics of Barley and Wheat Beer

**DOI:** 10.3390/foods13213400

**Published:** 2024-10-25

**Authors:** Xiu Li, Kai Jiang, Yuhong Jin, Junhan Liu

**Affiliations:** 1 Key Laboratory of Food Processing Technology and Quality Control in Shandong Province, College of Food Science and Engineering, Shandong Agricultural University, Tai’an 271018, China; 2 Laboratory of Chemistry of Natural Molecules, Gembloux Agro-Bio Tech, University of Liége, Passage des déportés 2, B-5030 Gembloux, Belgium

**Keywords:** beer, wheat malt, protein molecular weight, surface hydrophobicity, foam property

## Abstract

Protein is an important component of beer, and its type, content and molecular weight directly affect the quality of beer, especially the foam quality of beer. Different brands of wheat beer and barley beer available in the market were used for this analysis. The differences in protein composition and foam performance between multi-sample barley and wheat beer were analyzed using sodium dodecyl sulfate-polyacrylamide gel electrophoresis and high-pressure size exclusion chromatography. Protein significantly influences beer quality, particularly its foam properties. Wheat beer (WB) has 9.52–84.10% more total protein content than barley beer (BB). The primary proteins in both beers are 6.9–20.1 kDa, with WB having 1.04 g/L more of this protein, 60.11% higher than that of BB. It is one of the main different proteins between WB and BB. WB also contains 66.67% more 20.1–32.4 kDa protein compared to BB. This is one of the main differences between WB and BB proteins. Both 6.9–20.1 kDa and 20.1–32.4 kDa proteins enhance beer viscosity and foam properties. Additionally, WB’s > 32.4 kDa protein content is 246.67% higher than BB’s, significantly improving beer hydrophobicity and foam performance. These protein differences are key factors in the superior foam quality of WB.

## 1. Introduction

Beer is one of the most widely consumed alcoholic beverages in the world [1] and its consumption is increasing. Global beer production was about 189 million kiloliters in 2022, with China’s beer production reaching 35.687 million kiloliters. According to the Chinese national standard (GB/T 17204-2021) [2], the amount of wheat malt used in wheat beer (WB) should not be less than 30%. German regulations stipulate that WB (Weissbier) should be brewed with at least 50% malted or unmalted wheat as an adjunct to barley malt [3].

Like other alcoholic beverages, beer is treated with caution by the medical community [4]. However, as a fermented beverage, beer also offers certain health benefits [5]. Hops and malt contribute significant amounts of polyphenols, minerals (such as calcium, iron, magnesium, phosphorus, potassium, zinc, manganese, and selenium), as well as vitamins to beer [6]. Marcos et al. found that moderate beer consumption is beneficial for cardiovascular health and metabolism [7].

Protein, as one of the nutrients in beer, directly affects beer’s taste, color, and foam stability [8]. Foam is one of the main characteristics by which consumers judge beer quality. Around 80–85% of the proteins in beer originate from malt [9]. During the brewing process, proteins undergo a series of changes, including degradation, glycosylation, acylation, and structural unfolding into the finished beer. The stability of beer foam depends on the interaction of many components, mainly proteins/peptides from malt and iso-α acids from hops [10,11]. Protein Z (with a molecular weight of 40 kDa), lipid transfer protein 1 (LTP1), hordein, and barley dimeric α-amylase inhibitor 1 (BDAI-1) have been identified as foam-positive proteins [12,13,14]. Hydrophobic polypeptides were also most effective in stabilizing beer foam [15], whereas lipids and higher levels of ethanol were foam-negative components [16].

There are relatively few systematic studies investigating the effect of proteins in WB on foam properties. The use of wheat malt as an auxiliary ingredient in brewing beer can enhance the viscosity of beer liquor and thus improve the stability of beer foam [17]. When the amount of wheat malt is too high (>40%), it may lead to the high viscosity and reduced filtration performance of beer during the brewing process [18,19]. The use of wheat with lower protein content (12.6%) as a cofactor is more likely to cause protein precipitation or turbidity in the beer, which is detrimental to the stability of foam [20]. Low molecular-weight and higher molecular-weight proteins, on the other hand, were favorable for the foam properties, turbidity, and alcoholic strength of WB [21,22]. In addition, wheat malt, especially rye malt, was found to have the advantage of containing a large number of foam-positive components [23]. Hu et al. found that barley proteins are the primary source responsible for beer foam formation, and that wheat proteins play a key role in stabilizing the foam in wheat beer [24]. It has also shown that the proteins in the brewing process can expand their structure, change the internal hydrophobic structural regions, and enhance their surface activity to maintain foam stability [25].

The type, amount, and size distribution of proteins during beer production are important for filtration, foam and turbidity stability, and the fermentability of beer. Compared to barley malt, wheat malt has a higher soluble protein content [26]. The differences in protein content and composition between wheat and barley malt, as well as the unique brewing process of WB, inevitably lead to differences in the protein composition of WB compared to barley beer (BB). At present, there are fewer in-depth studies on the comparative differences in the foam properties of WB and BB; the research on the protein fractions controlling the quality of WB needs to be further deepened.

In this study, the differences in protein composition and foam performance between BB and WB were analyzed using sodium dodecyl sulfate-polyacrylamide gel electrophoresis (SDS-PAGE) and high-pressure size exclusion chromatography (HPSEC). The correlation of protein fractions and foam performance were also analyzed to provide a theoretical basis for the subsequent quality control of WB.

## 2. Materials and Methods

### 2.1. Materials

The 16 commercial beer samples used in this study were purchased from local supermarkets and the Jingdong online mall in Tai’an, China. Six were BB and 10 were WB. The physical and chemical indices of beer samples are shown in Table 1. The protein molecular-weight marker, which was 6.5–200 kDa (200, 116, 97.2, 66.4, 44.3, 29, 20.1, 14.3, and 6.5 kDa), was purchased from Takara (Kusatsu, Shiga City, Japan). Protein Mw standards for HPSEC were purchased from GE Healthcare (Beijing, China) with a range of weight average molecular weights (Wamw) as follows: conalbumin (Wamw 75.0 kDa); ovalbumin (Wamw 44.0 kDa); carbonic anhydrase (Wamw 29.0 kDa); ribonuclease A (Wamw 13.7 kDa); and aprotinin (Wamw 6.5 kDa). All other reagents were of at least analytical grade.

### 2.2. Beer Quality Parameters Analysis

Beer quality parameters were determined according to Analytica-EBC Section 8 and Analytica-EBC Section 9 [27] using the following methods: original gravity (°P)–EBC methods 9.4; alcohol content (% *v*/*v*)–EBC method 9.2.1; real attenuation (%)–EBC method 9.5; color (EBC)–EBC method 9.6; and total nitrogen content (mg/100)mL–EBC method 9.9.1. The protein content was the total nitrogen content multiplied by a conversion factor (6.25). Briefly, original gravity (°P) and alcohol content were determined using the distillation method. The sample was distilled with a distillation apparatus, and the distillate was collected and concentrated. The specific gravity of the distillate was then measured to determine the alcoholic content. The color of the beer was measured using a beer colorimeter (CS-820, Hangzhou Color Spectrum Technology Co., Ltd., Hangzhou, China), while viscosity was assessed with an Austrian viscometer. Total nitrogen content was determined via the Kjeldahl method, involving digestion and distillation of the samples, followed by titration to quantify the total nitrogen.

### 2.3. Measurement of Foam Properties

The measurement method followed the procedure described by Hu et al., with some modifications [24]. A 20 mL (*V*_0_) aliquot of beer samples was degassed by sonication at 20 °C for 20 min and then poured into the graduated cylinder. Carbon dioxide was injected into the degassed beer for 30 s at a constant pressure of 0.1 MPa. Afterward, the total volume of expansion (*V*_1_), foam volume (*V*_2_), and foam disappearance time were recorded immediately. Ten measurements with an average standard deviation <3% were selected for one sample. The formula is as follows:

(1) $$\mathrm{Foam}\;\mathrm{volume}\;\mathrm{expansion}\;\mathrm{rate}\;(\%)=\frac{V_{1}-V_{0}}{V_{0}}\times 100\%$$

(2) $$\mathrm{Foaming}\;\mathrm{capacity}=\frac{V_{1}-{\;V}_{0}}{V_{0}-(V_{1}-V_{2})}$$

### 2.4. Molecular Weight Determination of Proteins

The SDS-PAGE experiment followed the method of Guo et al. [28]. Briefly, after preparing the separating and concentrating gels, the samples were mixed with a sample buffer and boiled in a water bath for 10 min. Following centrifugation, the supernatant was loaded onto a BGverMINI electrophoresis instrument (Beijing Baijing Biotechnology Co., Ltd., Beijing, China). Electrophoresis was conducted at 70 V, after which the gels were stained with Coomassie Brilliant Blue for 4 h and then decolorized using a decolorizing solution. Scanning was carried out with a Bio–Rad gel imager (Shanghai Bio-Life Medical Products, Shanghai Co., Ltd., Shanghai, China). Details regarding the preparation of the separating and concentrating gels can be found in Table A1 (Appendix A).

The HPLC experiment referred to the method of Li et al. [29]. Briefly, samples were filtered with 0.45 μm and 0.22 μm membranes (Millipore Co., Milford, MA, USA). Measurement conditions were as follows: Chromatographic column: TSK gel Super SW2000 (4.6 mm × 30 cm, 44 μm) (Tosoh Co., Ltd., Tokyo, Japan), detector (SPD-20AT, Shimadzu, Kyoto, Japan); mobile phase: 80% (*v*/*v*) 0.2 mol/L phosphate buffer (pH 7.0) containing 0.15 mol/L NaCl and 20% (*v*/*v*) acetonitrile; the column temperature was 25 °C; the flow rate was 0.2 mL/min; the injection volume was 20 μL; the analysis time of the sample was 50 min; the detection wavelength of SPD-20AT was 214 nm; and the chromatographic resolution was performed using GPC software (EcoSEC GPC System).

### 2.5. Beer Surface Hydrophobicity

The beer solution was diluted with 0.01 mol/L phosphoric acid buffer (pH = 7.0) with the mass concentration of protein ranging from 10–400 mg/L, i.e., 50 mg/L, 100 mg/L, 150 mg/L, 200 mg/L, 250 mg/L, 300 mg/L. Four milliliters of the sample solution were used to determine the fluorescence intensity of the sample solution as (F0) and fluorescence intensity (F1) after adding 20 μL of ANS solution to the sample using a fluorescence spectrophotometer at an excitation wavelength of 395 nm (slit on excitation side was 5 nm) and 475 nm emission wavelength (slit on fluorescence side was 5 nm). The difference between F1 and F0 is denoted as F2. The protein concentration is used as the abscissa; F2 is the ordinate, and the slope of the curve is the surface hydrophobicity index.

### 2.6. Statistical Analysis

All data represent the mean value of three measurements. Data were processed using SPSS Statistics 27. Analysis of variance (ANOVA/Tukey) was used to compare the difference in the mean value of beers BB and WB. The difference under *p* < 0.05 was considered to be significant. The correlation was analyzed by Pearson correlation (double-tailed test), and a correlation under *p* < 0.05 was considered to be significant.

## 3. Results and Discussion

### 3.1. Differences in Protein Content and Composition Between Commercially Available WB and BB

#### 3.1.1. Protein Content of Commercially Available WB and BB

**Figure 1:**
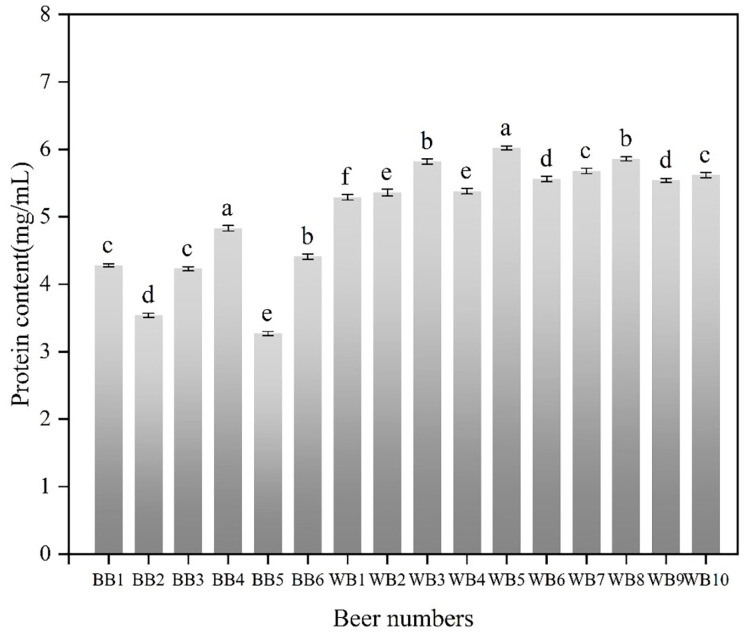
Protein content in commercial beers. BB, barley beer; WB, wheat beer. Different lowercase letters indicate a significant difference in the statistical analysis of the same type of beer (*p* < 0.05).

As shown in Figure 1, the protein content ranged from 3.27 mg/mL to 4.83 mg/mL in BB and from 5.29 mg/mL to 6.02 mg/mL in WB; the overall protein content of WB was significantly higher than that of BB, by 9.52% to 84.10%. The causes of this phenomenon are that the protein content of wheat (11.0–16.0%) is higher than that of barley (9.0–12.0%), and that the content of water-soluble polymer proteins in wheat is also higher than that in barley [26,30]. From the results of the correlation analysis in Table 2, it can be seen that the protein content in beer is significantly and positively correlated with beer foam stability, foam volume expansion rate, foam capacity, viscosity, and chroma. The correlation coefficients were 0.735, 0.831, 0.692, 0.690, and 0.725, respectively. This indicated that an increase in the total protein content of beer would improve the foam properties of beer.

#### 3.1.2. Molecular Composition of Proteins in Commercially Available WB and BB

**Figure 2:**
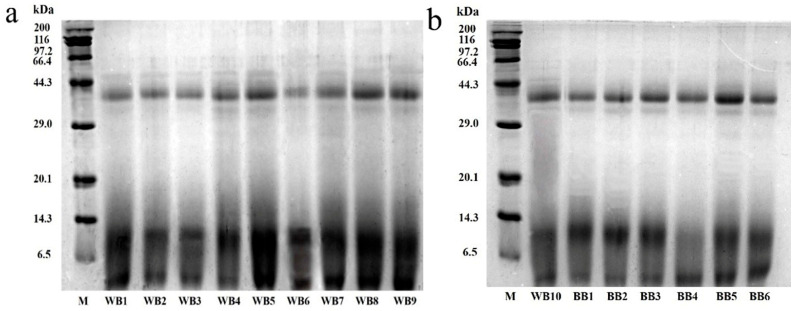
(**a**) WB1~WB9 protein molecular weight. (**b**) WB10,BB1~WB1 protein molecular weight. SDS-PAGE analysis of protein in commercial beers. M: protein standards, WB1~WB10: wheat beers 1~wheat beers 9, BB1~BB6: barley beer 1~barley beer 6.

SDS-PAGE was performed on 16 WB and BB; the molecular weight, relative content, and other information of the protein bands were analyzed by gel analysis software. From Figure 2, it can be seen that the distribution of the major bands of barley and WB proteins is relatively similar: protein bands were found at 40 kDa, 6.5–14.3 kDa, and less than 6.5 kDa. The results of the BB analysis indicated that around 40 kDa was protein Z and around 9 kDa was LTP [31].

The color intensity of the electrophoretic bands can provide an indication of the protein content at the corresponding molecular weight [32]. The color intensity of the electrophoretic bands increased progressively with higher protein content. There were differences in the composition of WB protein bands from that of BB, mainly in, as follows: (1) the darker color of protein bands less than 14.3 kDa; the darker color of protein bands around 17 kDa (17 kDa may be the 15–32 kDa residue of hordein that was reported to be associated with turbidity in beer [33,34] and it has been shown that 17 kDa of hordein was beneficial for beer foam [35]); (2) WB had lighter-colored protein bands between 20–32 kDa, and at 45 kDa and 66 kDa, that differed from those of BB. This means that WB has a lower content of these proteins; and (3) WB contained more small-molecule proteins <14.3 kDa and high molecular-weight proteins >45 kDa. This means that WB has a higher content of these proteins.

#### 3.1.3. Molecular Composition of Proteins in Commercially Available WB and BB and Its Correlation to the Physicochemical Properties of Beer

**Figure 3:**
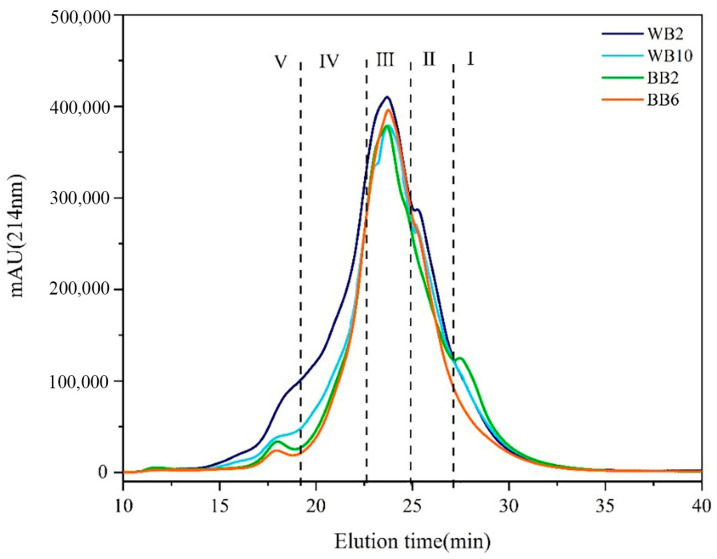
The molecular-weight distribution of proteins in commercial beers. Two samples of wheat beer and two samples of barley beer were selected to represent the molecular-weight spectra of all samples. I (MV < 1.1 kDa), II (MV 1.1–6.9 kDa), III (MV 6.9–20.1 kDa), IV (MV 20.1–32.4 kDa), V (MV > 32.4 kDa).

The molecular-weight composition of proteins in WB and BB is shown in Figure 3; separation of the proteins was based on their molecular weights and the chromatograms were classified into the following five different regions according to the peak time and peak shape: component I (<1.1 kDa); component II (1.1–6.9 kDa); component III (6.9–20.1 kDa); component IV (20.1–32.4 kDa); and component V (>32.4 kDa). The contents of the five components are listed in Table 3. The correlation analysis for the protein fractions of beer and the physicochemical indexes of beer are listed in Table 2.

The average content of <1.1 kDa protein component (component I) was 0.31 g/L in BB and 0.40 g/L in WB, which accounted for 9.79% and 8.07% of the total protein content of beer, respectively, with no significant difference in contents. The content of this component did not have a significant effect on the viscosity, chroma, surface hydrophobicity, foam stability, foam volume expansion rate, and foaming capacity of beer (*p* > 0.05).

The average content of 1.1–6.9 kDa protein component (component II) in BB and WB was 0.74 g/L and 0.49 g/L, accounting for 23.77% and 9.99% of the total protein content of the beers, respectively. The content of this protein in BB was 13.78% higher than that in WB. This part of the protein showed a significantly negative correlation (*p* < 0.05) with viscosity, chroma and foam stability, foam volume expansion rate, and foaming capacity, which might be due to the high solubility and mobility of the small-molecule proteins and polypeptides in beer. When beer contained higher concentrations of small molecular proteins and peptides, they dispersed with each other and formed a tighter bond with water molecules, which reduced the viscosity of beer and could penetrate through the bubble film, causing the bubbles to rupture and collapse rapidly [36,37].

The average content of the 6.9–20.1 kDa protein component (component III) in BB and WB were 1.73 g/L and 2.77 g/L, respectively, accounting for 55.05% and 55.82% of the total protein content of beer, which was the main protein in beer. Although the proportion of this protein was comparable between BB and WB, the content of WB was 1.04 g/L or 60.11% higher than that of BB, which was one of the main different components between WB and BB. This protein component showed a highly significant positive correlation (*p* < 0.01) with viscosity, chroma, foam stability, foam volume expansion rate, and foaming capacity. The significant positive correlation between viscosity and foam performance indicated that this protein component improved the foam performance of beer by increasing the viscosity of the beer system [38].

The average content of the 20.1–32.4 kDa protein component (component IV) in BB and WB was 0.21 g/L and 0.77 g/L, respectively, accounting for 6.50% and 15.60% of the total protein content in the beer. This component was 0.56 g/L higher in content and 66.67% higher in proportion in WB than in BB. This part of the protein was also one of the main different components between WB and BB. The positive correlation between this component and viscosity, chroma, foam stability, foam volume expansion rate, and foaming capacity was also highly significant, indicating that this component also improved the foam performance by increasing the viscosity of the beer system.

The average content of the >32.4 kDa protein component (component V) was 0.15 g/L and 0.52 g/L in BB and WB, respectively, accounting for 4.84% and 10.57% of the total protein content of beers. This component in WB was 0.37 g/L higher in content and 246.67% higher in proportion than that in BB. This component was the most significant differential protein between WB and BB. The significant positive correlation of this component with surface hydrophobicity and the highly significant positive correlation with viscosity and foam stability, foam volume expansion rate, and foaming capacity indicated that this component of the proteins mainly improved the hydrophobicity of beer and thus the foam properties [15].

### 3.2. Hydrophobicity Analysis of Commercially Available WB and BB

**Figure 4:**
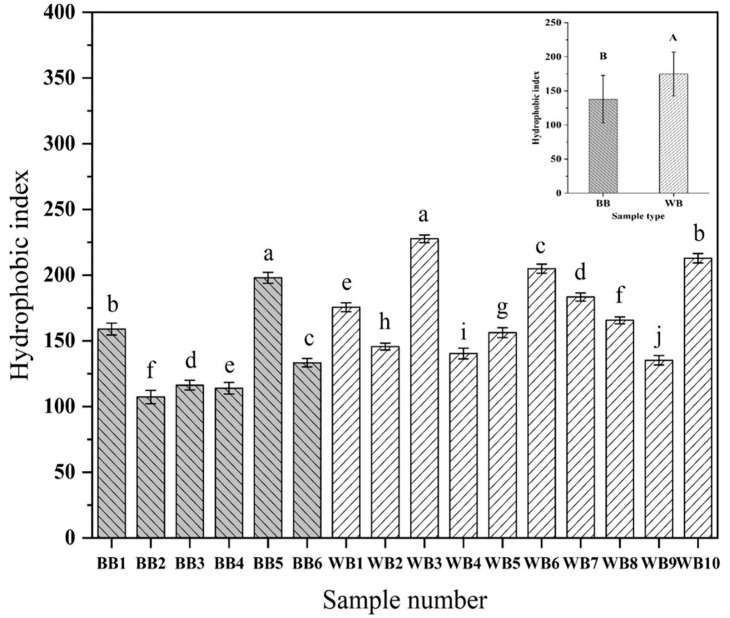
The surface hydrophobicity of commercial beer. Different lowercase letters indicate significant differences between the same type of beer. The chart at the top right shows the significant differences between the two types of beer. Different capital letters indicate significant differences between the two types beers (*p* < 0.05).

The hydrophobicity results of all commercially available beer samples were determined and are shown in Figure 4. The surface hydrophobicity indices of BB ranged from 107.22 to 198.00; in WB these ranged from 135.20 to 227.61. The average hydrophobicity of WB was significantly higher than that of BB (*p* < 0.05). Figure 1 demonstrates that the protein content in all WB samples was significantly higher than that in BB samples. However, Figure 4 indicates that the hydrophobicity of WB did not consistently exceed that of BB. This illustrates the fact that not all protein components affected the hydrophobicity of beer. This should be related to the number of hydrophobic groups in the protein molecule and the exposure of hydrophobic groups [39]. As exhibited in Table 3, the >32.4 kDa protein components in beer showed a significant positive correlation with the hydrophobicity of beer, while no correlation was found among the remaining protein components and the hydrophobicity of beer. This may be attributed to the presence of larger macromolecular proteins in beer, which contain more hydrophobic amino acid residues. The aggregation of these residues can lead to the formation of larger hydrophobic regions, resulting in enhanced hydrophobicity [40].

### 3.3. Principal Component Analysis of Protein Component Content and Physical and Chemical Indexes of Beer

The quality of beer cannot be determined by a single indicator alone. There was no obvious primary and secondary difference between the quality indicators; thus, it was not scientific to evaluate the quality of beer only based on a single indicator or a few correlated indicators [37]. The factor loadings and the eigenvalues and variances of the principal component analysis of the protein fraction content and the physicochemical indexes of beer in the commercially available beer are shown in Table 4. The 16 indicators were simplified into three principal components by principal component analysis; the cumulative variance contribution was 84.1%, and each principal component represented different protein components and physicochemical indicators in beer. The first principal component (PC1) explained 51.2% of the variance, indicating that PC1 played a dominant role in the analysis and evaluation. The indexes with higher loading values were chroma, foam stability, foam volume expansion rate, foaming capacity, total nitrogen, protein components II (1.1–6.9 kDa), III (6.9–20.1 kDa), IV (20.1–32.4 kDa), and V (>32.4 kDa) content. The indicators positively affected PC1 except for the protein component II content. The second principal component (PC2) explained 21.3% of the variance; the indicators with higher loading values were alcohol and original extract. The third principal component (PC3) explained 11.4% of the variance; the indicators with higher loading values were real degrees of fermentation.

**Figure 5:**
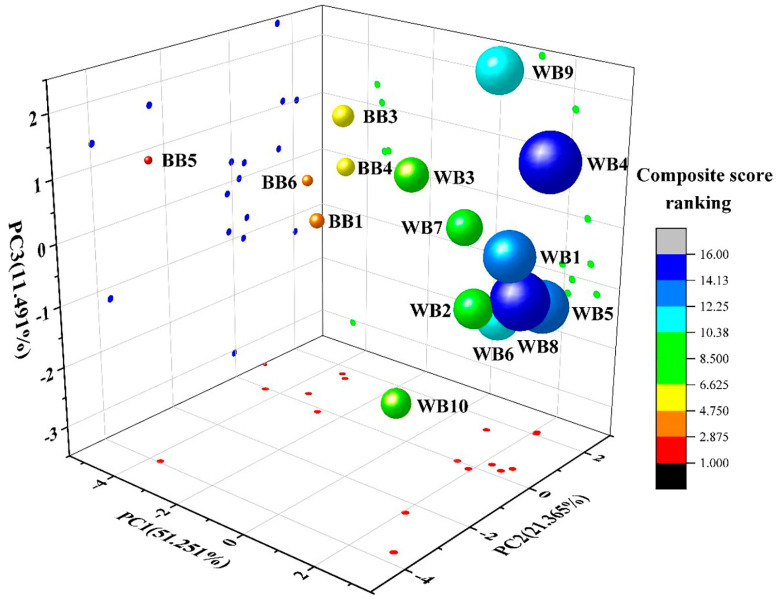
Distribution of PC1 vs. PC2 vs. PC3 scores of the main components of commercial beers.

Figure 5 shows the distribution of the composite scores of the commercially available beer samples based on principal component analysis. The higher the composite score of the principal components was, the better the quality of the beer was. As can be seen from Figure 5, along the direction of the PC1 axis, all the samples were gathered into two clusters, with BB in the left cluster and WB in the right cluster. This indicated that the difference in quality between BB and WB was mainly in the dominant PC1 variable, with higher loading values of >6.9 kDa protein components (components III, IV, and V) as well as foam stability, foaming capacity, and foam volume expansion rate. From the composite scores, all the WB composite scores were above 6.625, but all the BB composite scores were below 6.625, demonstrating that all the WB composite scores were higher than those of the BB. Combined with the analysis of 3.1 and 3.2, it was demonstrated that WB improved the foam performance of WB due to its higher content of >6.9 kDa protein, which led to the better quality of the WB.

## 4. Conclusions

The total protein content of WB was 9.52–84.10% higher than that of BB. Both 6.9–20.1 kDa and 20.1–32.4 kDa proteins could increase the viscosity of the beer system; thus, the protein content can improve the foam properties of beer. It was demonstrated that the protein enrichment in foam depended not only on the protein itself, but also on the beer system. Specifically, the effect of system viscosity plays a role in this enrichment; greater viscosity leads to better enrichment effects. The >32.4 kDa protein component in WB was 246.67% higher than that in BB, and it was the most important different protein between WB and BB. This protein mainly improved the hydrophobicity of beer and as a result, it could increase the foam performance of beer.

## Figures and Tables

**Table 1:** Physicochemical indices of commercial beers.

Beer Number	Country	Alcohol (% *v*/*v*)	Original Extract (°P)	Real Extract (% m/m)	Real Degree of Fermentation (%)	Chroma (EBC)	Viscosity (mPa·s)	Foam Stability (s)	Foam Volume Expansion Rate (%)	Foaming Capacity
BB1	China	4.90 ± 0.01 c	11.3 ± 0.1 cd	3.81 ± 0.01 cd	67.6 ± 0.2 d	6.3 ± 0.0 a	1.41 ± 0.00 b	280 ± 3 b	21.67 ± 0.87 b	0.41 ± 0.04 b
BB2	Germany	4.50 ± 0.01 d	11.1 ± 0.0 d	4.21 ± 0.00 a	63.4 ± 0.0 e	5.2 ± 0.0 d	1.50 ± 0.01 a	220 ± 4 e	11.58 ± 0.78 f	0.22 ± 0.05 e
BB3	Germany	5.58 ± 0.01 a	12.3 ± 0.1 a	3.84 ± 0.01 c	70.2 ± 0.1 a	5.9 ± 0.0 b	1.42 ± 0.01 b	256 ± 6 c	16.89 ± 0.69 e	0.36 ± 0.07 d
BB4	Russia	5.32 ± 0.01 b	12.0 ± 0.3 b	3.93 ± 0.02 b	68.8 ± 0.1 b	5.1 ± 0.0 d	1.38 ± 0.00 b	250 ± 4 d	18.33 ± 0.42 d	0.39 ± 0.08 c
BB5	Russia	4.26 ± 0.02 e	9.8 ± 0.2 e	3.22 ± 0.01 e	68.2 ± 0.1 c	5.8 ± 0.0 bc	1.39 ± 0.01 b	296 ± 3 a	21.96 ± 0.48 a	0.45 ± 0.05 a
BB6	Germany	5.05 ± 0.02 c	11.5 ± 0.0 c	3.78 ± 0.00 d	68.5 ± 0.1 bc	5.6 ± 0.0 c	1.38 ± 0.01 b	260 ± 6 c	18.54 ± 0.74 c	0.37 ± 0.06 d
Mean (n = 6)		4.94 ± 0.49 A	11.3 ± 0.9 A	3.80 ± 0.32 A	67.8 ± 2.3 A	5.7 ± 0.5 B	1.41 ± 0.05 B	260 ± 26 B	18.16 ± 3.79 B	0.37 ± 0.08 B
WB1	Germany	5.17 ± 0.04 c	11.9 ± 0.2 bc	3.99 ± 0.01 c	67.7 ± 0.2 d	9.4 ± 0.0 c	1.70 ± 0.01 abc	310 ± 4 e	38.46 ± 0.57 e	0.80 ± 0.08 b
WB2	Germany	4.84 ± 0.00 e	11.4 ± 0.0 d	3.99 ± 0.01 c	66.3 ± 0.1 f	10.3 ± 0.0 b	1.65 ± 0.01 d	300 ± 6 f	39.17 ± 0.68 d	0.79 ± 0.10 b
WB3	Germany	4.63 ± 0.01 f	10.6 ± 0.0 e	3.46 ± 0.01 f	68.5 ± 0.0 c	7.4 ± 0.0 i	1.68 ± 0.00 cd	370 ± 2 a	46.87 ± 0.52 a	0.82 ± 0.01 a
WB4	Germany	5.61 ± 0.01 a	12.5 ± 0.1 a	4.00 ± 0.00 c	69.5 ± 0.0 b	7.5 ± 0.0 h	1.59 ± 0.01 e	345 ± 5 b	43.33 ± 0.74 b	0.74 ± 0.01 d
WB5	Germany	5.39 ± 0.00 b	12.5 ± 0.0 a	4.36 ± 0.00 a	66.7 ± 0.0 e	9.2 ± 0.0 d	1.72 ± 0.01 ab	304 ± 3 f	31.67 ± 0.82 g	0.47 ± 0.02 i
WB6	Germany	5.02 ± 0.00 d	11.8 ± 0.0 cd	4.14 ± 0.01 b	66.3 ± 0.2 f	8.9 ± 0.0 e	1.73 ± 0.01 a	338 ± 5 c	33.33 ± 0.64 f	0.51 ± 0.04 h
WB7	Germany	5.17 ± 0.00 c	11.9 ± 0.0 bc	3.99 ± 0.00 c	67.7 ± 0.0 d	8.2 ± 0.0 g	1.48 ± 0.00 f	316 ± 4 d	38.42 ± 0.71 e	0.58 ± 0.07 g
WB8	China	5.02 ± 0.00 d	11.8 ± 0.0 cd	4.15 ± 0.00 b	66.2 ± 0.0 f	8.6 ± 0.0 f	1.69 ± 0.00 bc	350 ± 4 b	41.27 ± 0.63 c	0.66 ± 0.07 e
WB9	China	5.68 ± 0.01 a	12.3 ± 0.2 ab	3.65 ± 0.00 e	71.6 ± 0.1 a	6.9 ± 0.0 j	1.60 ± 0.02 e	320 ± 6 d	38.38 ± 0.51 e	0.62 ± 0.0 f
WB10	China	4.19 ± 0.01 g	10.3 ± 0.1 e	3.88 ± 0.02 d	63.7 ± 0.1 g	10.8 ± 0.0 a	1.44 ± 0.01 g	368 ± 6 a	43.33 ± 0.43 b	0.77 ± 0.05 c
Mean (n = 10)		5.07 ± 0.45 A	11.7 ± 0.7 A	3.96 ± 0.26 A	67.4 ± 2.1 A	8.7 ± 1.3 A	1.62 ± 0.10 A	332 ± 25 A	39.421 ± 4.59 A	0.68 ± 0.13 A

BB, barley beer; WB, wheat beer. Mean represents the average value of the same type of beer sample, different capital letters in the same column indicate significant differences between different types of beer, and different lowercase letters indicate significant differences between the same type of beer in the same column (*p* < 0.05).

**Table 2:** Correlation analysis of protein component content and physicochemical indexes in beer.

Beer Index	Protein Component Content					Viscosity	Chroma	Protein Content	Surface Hydrophobicity	Foam Stability	Foam Volume Expansion Rate
	<1.1 kDa	1.1–6.9 kDa	6.9–20.1 kDa	20.1–32.4 kDa	>32.4 kDa						
Viscosity	0.466	−0.534 *	0.797 **	0.780 **	0.463	1					
Chroma	0.310	−0.767 **	0.675 **	0.829 **	0.827 **	0.618 *	1				
Protein content	0.356	0.421	0.947 **	0.912 **	0.696 **	0.690 **	0.725 **	1			
Surface hydrophobicity	−0.067	−0.493	0.378	0.344	0.498 *	0.146	0.357	0.377	1		
Foam stability	0.313	−0.622 **	0.708 **	0.668 **	0.687 **	0.542 *	0.685 **	0.735 **	0.666 **	1	
Foam volume expansion rate	0.383	−0.637 **	0.782 **	0.807 **	0.777 **	0.640 **	0.762 **	0.831 **	0.548 *	0.927 **	1
Foaming capacity	0.370	−0.601 **	0.677 **	0.682 **	0.756 **	0.552 *	0.743 **	0.692 **	0.497 *	0.830 **	0.940 **

Data were analyzed by Pearson correlation (double-tail *t*-test), sample size n = 16, * indicating significant correlation at *p* < 0.05 level, ** indicating extremely significant correlation at *p* < 0.01 level.

**Table 3:** Absolute content of protein components in beers (g/L).

	I (MV < 1.1 kDa)	II (MV 1.1–6.9 kDa)	III (MV 6.9–20.1 kDa)	IV (MV 20.1–32.4 kDa)	V (MV > 32.4 kDa)
	0.45 kDa	2.64 kDa	12.52 kDa	28.06 kDa	68.94 kDa
BB1	0.47 ± 0.06 a	0.76 ± 0.05 d	1.94 ± 0.05 b	0.20 ± 0.01 c	0.17 ± 0.08 b
BB2	0.35 ± 0.08 b	0.78 ± 0.08 c	1.65 ± 0.07 d	0.11 ± 0.02 d	0.16 ± 0.05 bc
BB3	0.25 ± 0.07 d	0.68 ± 0.02 e	1.52 ± 0.09 e	0.29 ± 0.08 a	0.10 ± 0.02 e
BB4	0.31 ± 0.02 c	0.81 ± 0.05 b	2.15 ± 0.10 a	0.23 ± 0.03 b	0.15 ± 0.04 cd
BB5	0.17 ± 0.05 e	0.58 ± 0.02 f	1.27 ± 0.07 f	0.10 ± 0.07 d	0.14 ± 0.08 d
BB6	0.33 ± 0.08 bc	0.86 ± 0.04 a	1.89 ± 0.08 c	0.31 ± 0.09 a	0.19 ± 0.06 a
Mean	0.31 ± 0.10 A	0.74 ± 0.10 A	1.73 ± 0.32 B	0.21 ± 0.09 B	0.15 ± 0.03 B
WB1	0.43 ± 0.09 c	0.45 ± 0.02 f	2.40 ± 0.09 i	0.81 ± 0.08 c	0.72 ± 0.01 b
WB2	0.43 ± 0.02 c	0.54 ± 0.07 c	2.63 ± 0.07 f	0.80 ± 0.06 cd	0.45 ± 0.02 e
WB3	0.33 ± 0.01 e	0.66 ± 0.03 a	2.94 ± 0.04 c	0.60 ± 0.08 h	0.41 ± 0.06 f
WB4	0.63 ± 0.06 a	0.47 ± 0.07 e	2.74 ± 0.03 d	0.72 ± 0.09 f	0.38 ± 0.09 g
WB5	0.33 ± 0.07 e	0.56 ± 0.02 b	3.15 ± 0.08 a	1.02 ± 0.08 a	0.45 ± 0.07 e
WB6	0.48 ± 0.08 b	0.52 ± 0.04 d	3.01 ± 0.10 b	0.75 ± 0.03 e	0.41 ± 0.08 f
WB7	0.25 ± 0.09 f	0.42 ± 0.07 g	2.72 ± 0.08 e	0.84 ± 0.04 b	0.50 ± 0.04 d
WB8	0.42 ± 0.04 c	0.44 ± 0.01 f	3.16 ± 0.06 a	0.79 ± 0.05 d	0.38 ± 0.06 g
WB9	0.36 ± 0.02 d	0.48 ± 0.03 e	2.52 ± 0.05 g	0.71 ± 0.07 f	0.60 ± 0.03 c
WB10	0.34 ± 0.04 e	0.41 ± 0.02 g	2.44 ± 0.06 h	0.67 ± 0.07 g	0.89 ± 0.02 a
Mean	0.40 ± 0.10 A	0.49 ± 0.08 B	2.77 ± 0.28 A	0.77 ± 0.11 A	0.52 ± 0.17 A

MV, average molecular weight; BB, barley beer; WB, wheat beer. The difference analysis of the same type of beer was carried out in one group, and different lowercase letters in the same group in the same column indicated a significant difference (*p* < 0.05). Mean represents the average value of the same type of beer sample, and capital letters indicate significant differences among different types of beer (*p* < 0.05).

**Table 4:** Factor loadings of variables and evaluating indices of eigenvalues and variances.

	PC1	PC2	PC3
Alcohol	0.109	**0.909**	0.377
Original extract	0.199	**0.965**	0.072
Real extract	0.295	0.593	−0.733
Real degree of fermentation	−0.128	0.402	**0.901**
Chroma	**0.880**	−0.164	−0.268
Viscosity	0.773	0.229	−0.137
Surface hydrophobicity	0.552	−0.705	0.123
Foam stability	**0.860**	−0.319	0.255
Foam volume expansion rate	**0.940**	−0.151	0.233
Foaming capacity	**0.848**	−0.237	0.269
Protein content	**0.907**	0.225	0.054
Protein component I	0.448	0.402	−0.165
Protein component II	**−0.821**	0.125	−0.067
Protein component III	**0.883**	0.246	−0.087
Protein component IV	**0.931**	0.245	−0.040
Protein component V	**0.827**	−0.203	−0.039
Eigenvalue	8.200	3.418	1.839
Explanatory variance	51.251	21.365	11.491
Cumulative variance	51.251	72.617	84.108

PC1, the first principal component; PC2, the second principal component; PC3, the third principal component. Factor load values above 0.80 are indicated by bold numbers.

## Data Availability

The original contributions presented in the study are included in the article, further inquiries can be directed to the corresponding author.

## Appendix A

**Table A1:** Preparation ratio of separating gel and concentrated gel.

Reagents	Unit	15% Separating Gel	6% Concentrated Gel
ddH_2_O	mL	1.65	3.50
30% Acr-Bis (29:1)	mL	7.50	0.84
1.0 mol/L Tris-HCl pH = 8.8	mL	5.60	0
1.0 mol/L Tris-HCl pH = 6.8	mL	0	0.625
10% SDS	μL	150	50
10% Ammonium persulfate	μL	75	50
TEMED	μL	10	5
